# An Autoencoder-Based Deep Learning Classifier for Efficient Diagnosis of Autism

**DOI:** 10.3390/children7100182

**Published:** 2020-10-14

**Authors:** Harshini Sewani, Rasha Kashef

**Affiliations:** Department of Electrical, Computer, and Biomedical Engineering, Ryerson University, Toronto, ON M5B 2K3, Canada; harshini.sewani@ryerson.ca

**Keywords:** autism, diagnosis, autoencoder, convolution neural network, machine learning

## Abstract

Autism spectrum disorder (ASD) is a neurodevelopmental disorder characterized by a lack of social communication and social interaction. Autism is a mental disorder investigated by social and computational intelligence scientists utilizing advanced technologies such as machine learning models to enhance clinicians’ ability to provide robust diagnosis and prognosis of autism. However, with dynamic changes in autism behaviour patterns, these models’ quality and accuracy have become a great challenge for clinical practitioners. We applied a deep neural network learning on a large brain image dataset obtained from ABIDE (autism brain imaging data exchange) to provide an efficient diagnosis of ASD, especially for children. Our deep learning model combines unsupervised neural network learning, an autoencoder, and supervised deep learning using convolutional neural networks. Our proposed algorithm outperforms individual-based classifiers measured by various validations and assessment measures. Experimental results indicate that the autoencoder combined with the convolution neural networks provides the best performance by achieving 84.05% accuracy and Area under the Curve (AUC) value of 0.78.

## 1. Introduction 

Autism spectrum disorder (ASD) is one of the brain development disorders. According to the World Health Organization (WHO), 1 in 60 children has an autism spectrum disorder [1]. Difficulties in social communication and interaction characterize this disorder; individuals on the spectrum also tend to have restricted interest and repetitive behaviour. ASD is an intellectual disability; many of those on the autism spectrum have extraordinary abilities and skills. Roughly 40% are intellectually above average and have a unique ability to see the world with pride from a different perspective. According to the National Autism Spectrum Disorder Surveillance System (NASS), the most up-to-date Canadian prevalence rate is: 1 in 66 Canadian children and youth (ages 5–17) were diagnosed with autism spectrum disorder [2]. According to the National Institute of Mental Health (NIHM) [3], scientists do not know how and what causes autism, but some research suggests that genes and environmental factors cause autism. Some of the potential risk factors associated with ASD include having a sibling with ASD, older parents, pre-existing genetic conditions like Down syndrome, fragile X syndrome, and Rett syndrome, and finally, a low birth weight. Additionally, it is worth mentioning that researchers found differences in the brains of babies born before 27 weeks [4], i.e., babies born very prematurely are at higher risk for developing ASD. Early diagnosis in the first few years of life significantly improves results for people on the autism spectrum, but there are often delays in recognizing and diagnosing ASD. If health systems were better capable of identifying children at high risk for ASD and bringing them in earlier for a comprehensive evaluation, more children could benefit from early intervention. Many machine learning and neural network methods have recently shown an improvement in autism diagnosis [4,5,6,7]. The classification accuracy and the required computational time for building an automatic diagnosis system are significant challenges for many classifiers. In the paper, we focus on classifying individuals who have ASD from typically developing controls subjects using functional magnetic resonance imaging (fMRI) images provided by the autism brain imaging data exchange (ABIDE) [8] to study brain activities. There are 17 different brain imaging centers from where these images are collected. The dataset contains 539 individuals who have ASD and 573 typical controls (TC). ABIDE is preprocessed by four different pipelines like the connectome computation system (CCS), the configurable pipeline for the analysis of connectomes (CPAC), the data processing assistant for resting-state fMRI (DPARSF), and the neuroimaging analysis kit. We used the CPAC pipeline with preprocessing steps, including slice time correction, motion correction, nuisance signal remover, low-frequency drift, and voxel intensity normalization. We propose a hybrid classifier that combines an autoencoder with some well-known supervised machine learning algorithms and deep learning methods. For the deep learning methods, we focused on the convolution neural network (CNN). The autoencoder, combined with CNN, has shown a maximum accuracy of 83.39%. This paper is structured as follows: In Section 2, a literature survey is provided. Section 3 discusses the algorithms used from the machine learning and the neural network literature and essential parameters in each algorithm. In Section 4, we introduced the autoencoder-based hybrid diagnosis model. Section 5 provides details on the performance of the individual and hybrid algorithms using rs-fMRI experimental datasets. In Section 6, we concluded the paper along with some future directions.

## 2. Literature Review

Various machine learning and deep neural network methods distinguish between ASD and non-ASD. These methods are categorized into three main categories as image-based, questionnaire-based, or behavioural-based.

### 2.1. Image-Based Classification

Several studies have employed deep neural network learning on large brain image datasets obtained from the autism brain imaging data exchange [8] to identify ASD individuals from the typical control (TC). In [4], the authors used the deep learning model to perform binary classification of ASD and the typical control based on their neural patterns using rs-fMRI data. There were 505 ASD participants and 530 typically developing controls from 17 different imaging sites. They opted for using two stacked autoencoders to extracted lower-dimensional features. The model achieved 70% accuracy, a sensitivity of 74%, and a specificity of 63%, which is better than support vector machines (SVMs) and random forest (RF) models used in a previous study; these models used 10-fold cross-validation. Due to the high probability of noise in the image dataset obtained from several sites, the model does not achieve promising results despite using a dedicated Graphical Processing Unit (GPU) to speed up the training time. The entire model took about 33 h for training. Another model that diagnosed ADHD and autism from 3-D structural MRI and 4-D (fMRI) is presented in [9]. There are three learners in their work, including texture-based filters obtained using the sparse encoders that extracts features from MRI datasets. The fMRI scans are used to compute spatial non-stationary independent components, which decompose the subject’s scans into the sequence order in time from the obtained component. The multimodal features obtained from the learner serves as input to the SVM model. The accuracy of the ADHD-200 dataset is 67.3%, and the accuracy of the ABIDE dataset is 64.3%. These learning models have found a “Signal” in the data to identify differences between case and control. The current results are not yet clinically applicable. In [10], authors have used 6 personal characteristic data (PCD) of 851 subjects (421 ASD and 430 non-ASD) from the ABIDE database to predict autism. The authors have evaluated the performance of nine machine learning models. Comparing to the other eight models, the neural network-based stacked sparse encoder performance is better with an area under the curve of 0.646. The advantage of their work includes understanding the predictive power of PCD for ASD classifications. The limitation of their work was that the dataset is from 17 different clinical and research sites; this fact leads to heterogeneity in the data and might underfit the models’ accuracy; the database’s size is another issue. In [5], a hybrid approach is introduced to detect autism using fMRI data [5]. In their work, they have proposed the ASD-DiagNet framework. Their experimental dataset is preprocessed using the C-PAC pipeline and parcellated into 200 functionally regions. They have used data augmentation techniques using the synthetic minority over-sampling technique (SMOTE). SMOTE uses the nearest neighbour method to generate augmented data. The process is performed using two phases; the first phase evaluates the model’s performance on the whole dataset to find functional connectivity between regions, which is acquired using Pearson’s correlation. The results achieved an accuracy of 69.4% on the original dataset, whereas data generated using augmentation shows some improvement, i.e., 70.3% accuracy. The second phase of their work includes 5-fold cross-validation on every 17 sites separately. The average result of all sites with the ASD-DiagNet model without augmented data is 60.7% accuracy, and 63.8% with augmented data. Another experiment was performed on automated anatomical labelling (AAL) and Talairach and Tournoux (TT). For AAL, 67.5% is the highest accuracy obtained (using augmented data), whereas TT achieved an accuracy of 65.3% (using augmented data). The convolutional neural network has been applied to detect the autism spectrum disorder using the ABIDE dataset [11] and has achieved 70.22% accuracy using fewer parameters. The authors pointed out four essential regions for ASD classification: C115, C188, C247, and C326 for the CC400 functional parcellation. The performance of SVM, K-nearest neighbour (KNN), and the random forest was evaluated, and achieved accuracy 69%, 62%, and 60%, respectively, after hyperparameter tuning. Their work has used fewer parameters, which helped in the reduction of computational cost.

### 2.2. Questionnaire-Based Classification Methods

In [12], a research study had attempted to detect ASD and ADHD using a crowdsource recruitment procedure. The survey database contains 248 (ASD) and ADHD (174) subjects, aged 2–17. The second dataset collected from the previous study is called archival, including 2775 ASD and 150 ADHD. Five machine learning models, ENet, Lasso, SVM [13], linear discriminant analysis (LDA) [14], and Ridge, were used. These models are applied to three independent datasets and combinations of the archival and survey datasets. All five models achieved Area under the Curve (AUC) that exceeds 0.90 when the archival dataset was used as training. When considering the survey sample as training, the ENet and LDA models work well. By using the mixture of two datasets, they have achieved an AUC equal to 0.89. The disadvantages are that the data obtained in archival ADHD is composed of siblings of children with autism. This data is biased, and therefore, results are compromised. In [15], authors have used two algorithms to train a structure parent-reported questionnaires and significant behaviours from short videos of children [15]. The dataset is obtained from multiple repositories of Autism Diagnostic Observation Schedule (ADOS) and Autism Diagnostic Interview Revised (ADI-R) score-sheets of children between 18 and 84 months. The random forest (RF) is trained over the ADI-R instrument data on a parent questionnaire (2299 with ASD, 585 with TC, and 364 with other conditions). The video of the subject (i.e., a 1-min home video taken by parents) was required to evaluate the target label’s presence. The responses of all the questionnaires and short clips collaborated using L2-regularized logistic regression. The Receiver Operating Characteristics (ROC) of the combined data shows a boost in the performance of the clinical study samples. The advantage is that the ROC curve outperforms when compared with tools like Modified Checklist for Autism in Toddlers (M-CHAT) and Child Behaviour Checklist (CBCL). Additionally, allowing some subjects with lower certainty output from the algorithms to be classified as inconclusive. Their work utilizes experts who have used ADI-R and ADOS tools. These tools consumed hours to evaluate the results since parents were conducting it without experts and not more than a minute to complete the test, which caused significant data degradation and adding bias with an expected loss of screening accuracy. The authors have utilized machine learning algorithms for detecting autism in the below-explained study [16]. They have used three ASD datasets available from the University of California Irvine (UCI) repository (https://archive.ics.uci.edu). The dataset has 20 attributes, of which 1–10 are screening questions, and the remaining 10 attributes are personal information. The dataset has missing values, which were handled during the preprocessing steps before applying machine learning models. They have evaluated SVM, random forest, and KNN by splitting the dataset into five different sets. The results show that the random forest has performed very well for the classification compared to SVM and KNN. The authors recommend in their study to use a large data set and fewer missing values. Authors have worked on the UCI dataset [17] to predict autism using machine learning and neural network models [18]. The accuracy of the CNN and SVM models on the adult dataset exceeds 98%, while CNN’s accuracy on the youth data set exceeds 96%. The CNN, Artificial Neural Networks (ANN), logistic regression, and SVM achieve accuracy above 98% on the children dataset.

### 2.3. Behavioural-Based Classification Methods

In [19], the authors identified a few behavioural measures that were enough to differentiate ASD from ADHD. The data set is from the Boston Autism Association and Autism Genetic Resources Exchange of 2775 autistic individuals and 150 ADHD subjects. Using Social Responsiveness Scale (SRS) items containing responses of a child’s behaviour, six machine learning models like SVM, LDA, categorical Lasso, and logistic regression, random forest, and decision tree was trained and tested. Minimal redundancy, maximal relevance, undersampling, forward feature selection, and 10-fold cross-validation applied to the dataset before training. The results show out of six algorithms, four algorithms, including SVM, LDA, categorical Lasso, and logistic regression, achieved an accuracy ranging from 96.2% to 96.5%. Each model used only 5 of the 65 behaviour indicators. The models mentioned were able to do classification tasks optimally, not only due to less error but probabilistic qualities. The disadvantages of their work include the massive imbalance between subjects of ASD and ADHD. This subject imbalance was overcome with undersampling but prevented authors from devoting a part of data exclusively for validation because of the constrained sample data. In [20], authors have designed a normalization layer and activation layer into a single tensor to tensor computation graph, which forms high sparse and large search space. The normalization layer and activation function are essential components of deep learning that stabilize optimization and improved generalization. The authors have shown an experiment of Evo Norms (normalization-activation layer) on image classification models such as Res Nets, Mobile Nets, and Efficient Nets. Evo Norms consist of two series: (1) the B-series, which is batch dependent, and (2) the S-series, which works on independent samples introduced by rejecting any batch dependent operation. Their method discovered novel layers with structures that achieved strong generalization across many architectures and tasks.

## 3. Machine Learning and Deep Learning Classifiers

In our proposed hybrid model, we combined an autoencoder for dimensionality reduction with one of the well-known classifiers, including support vector machines (SVMs), random forest (RF), K-nearest neighbours (KNNs), and convolutional neural network (CNN). This section introduces each of the adopted methods in our hybrid model.

### 3.1. Autoencoder

The autoencoder is a type of neural network that does not require the labeling of data, and therefore it is an unsupervised learning algorithm. The aim is to learn an input function to reconstruct the input to an output of fewer dimensions. It approximates the identity function to get the outcome of a neural network similar to the input. In order words, it tries to copy the input to its output. Mathematically, if *x* is the input (also called an encoder), *x*’ is the network (also called a decoder). The architecture of autoencoders reduces dimensionality using non-linear optimization. In Equation (1), *h* is the hidden layer, which can be calculated by multiplying the vector *x* with weights and adding the bias and passing it to the activation function. The decoder *x*’ is calculated in Equation (2).

(1) h=σ(W×x+b)

(2) $$x^{\prime }=\left(W\times h+b\right)$$

There are many autoencoder variants, such as the undercomplete autoencoder, denoising autoencoder, sparse autoencoder, and adversarial autoencoder. A research study in [21] has demonstrated that when the input has a relation or a basic structure, the input reconstruction will be painless. They have described several applications that involve the use of the autoencoder algorithm, one of which is brain disorders. Whenever clinical neuroimaging studies are considered, they always opt for an autoencoder before feeding data to any other classification model because of the high dimensionality of genetic and neuroimaging data. The difficulty lies in constructing and learning output from a high dimensional input. In [22], the authors have evaluated the performance by comparing an autoencoder with other models that can do a similar task of reduction, for example, models like principal component analysis, linear discriminant analysis (linear models), locally linear embedding, and Isomap (non-linear models). They have applied their model on various datasets and concluded that the number of hidden layer nodes affects the autoencoder’s performance. When the hidden nodes adjusted around the dataset’s inherent dimensionality, the Modified National Institute of Standards and Technology (MNIST) data set performs well. Other experiments in their paper involved a synthetic dataset and Olivetti dataset. Experimental analysis on the synthetic dataset showed stable performance by principal component analysis (PCA), linear discriminant analysis (LDA), and Isomap. However, an autoencoder and locally linear embedding (LLE) were not stable. The unstable models retain the original data’s geometric characteristics, so the result is still acceptable. Experiments on the MNIST dataset showed that the autoencoder performed better than PCA. The autoencoder projected points to represent the digit “1”. An autoencoder tends to project images of the same class to edges and corners. The results showed that the autoencoder not only helps in dimensionality reduction but also in detecting the recurrent structures. The lower-dimensional data generated during the encoding process contains useful patterns from the original input, is provided to a convolutional neural network or a machine learning algorithm [23,24,25].

### 3.2. Support Vector Machines (SVMs)

**Figure 1:**
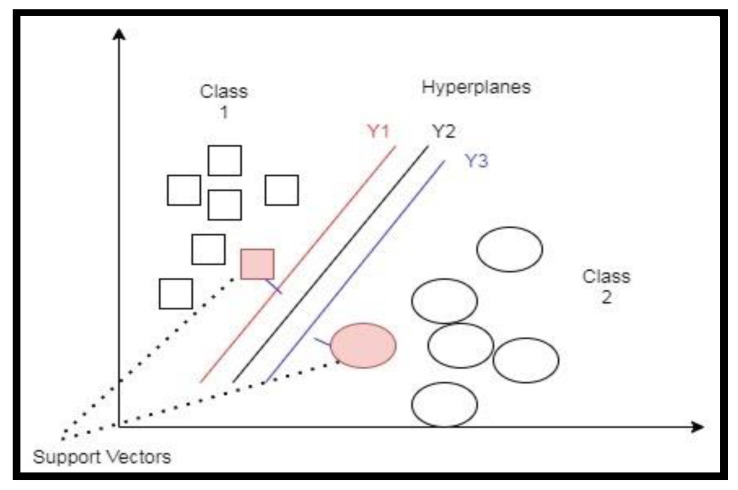
Hyperplane of the support vector machine (SVM).

Support vector machines (SVMs) are a supervised machine learning model applied in regression and classification tasks. The SVM aims to find a hyperplane in an N-dimensional space that classifies the data points. Let us consider an example of two classes. Figure 1 shows multiple hyperplanes (Y1, Y2, and Y3) that divide these data points into their respective classes. We had to choose a hyperplane that assures data points are on the right side of the graph. From Figure 1, we could conclude that Y2 segregated data points more efficiently. Two points were closest to the two lines (Y1 and Y3) and had less margin. These points (pink filled shapes) were a little further from hyper-plane Y2. Hence, we set Y2 as the right hyperplane with a high margin.

In this paper, we perform binary classification such that the SVM predicts the binary outcome on brain image data. The knowledge of the SVM is incomplete without understanding kernels. Kernel functions play a vital role in SVM. They transform inputs space into feature space in any required form. Kernels provide shortcuts to avoid heavy calculations. The significant fact about the kernel is that we can go to higher dimensions and perform effortless calculations. With the kernels, we can go up to an infinite number of dimensions using kernels. There are various types of kernels; some of these kernels are discussed as follows: [26,27,28,29].

- Linear: This form of kernel function is very simple, straightforward. It is given by the inner product of (*x*,*y*) plus an optional constant bias, as shown in Equation (3):
  (3) $$k\left(x,y\right)=x^{T}y+bias$$
- Sigmoid: The sigmoid kernel is also called hyperbolic tangent kernel and as a multilayer perceptron kernel. The sigmoid kernel is obtained from the neural network field, where the bipolar sigmoid function is used as an activation function for the neurons.
  (4) $$k\left(x,y\right)\;=\;\propto \times \tan h\left(x^{T}y+bias\right)$$
- Radial basis function (RBF): is used when we have no prior knowledge of data.
  (5) $$k\left(x,y\right)=\exp\left(\frac{{\left|\left|x-y\right|\right|}^{2}}{2\sigma^{2}}\right)$$

The SVM is an effective classifier when the number of dimensions is greater than the number of data samples. SVM has high-efficiency memory functions and has various forms of kernels [13].

### 3.3. Random Forest

**Figure 2:**
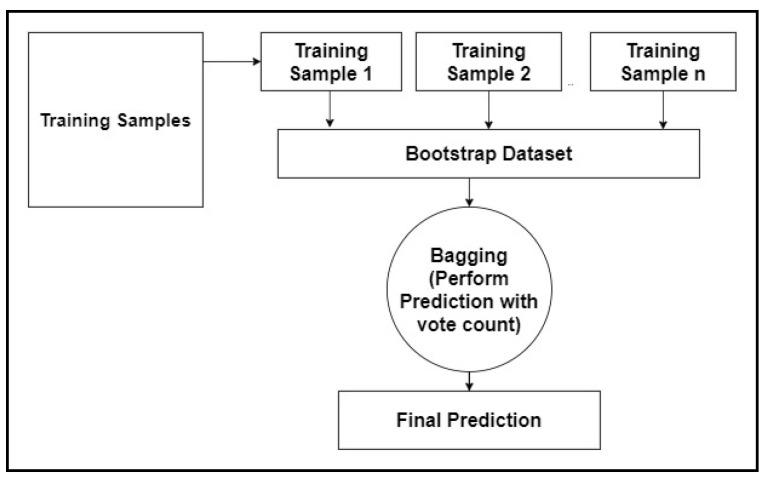
The random forest algorithm.

An ensemble of decision trees forms the random forest (RF). The disadvantage of using decision trees is that they are not flexible enough to classify new sets of data points. Random forest combines the simplicity of a decision tree with flexible results and improves performance metrics. The following steps are required to build a random forest tree, as in Figure 2.

- Step 1: Building decision trees using a bootstrap dataset.
- Step 2: Consider a random subset of variables at each step.
- Step 3: Perform a vote for a new dataset by sending it to all the trees.
- Step 4: Select the prediction result with the highest votes as the final prediction.

The bootstrap dataset does not select all columns to determine the root of the tree. Step 1 mentioned above is performed again until varieties of trees are built. The idea of creating multiple trees leads to an efficient performance of the random forest. Assume a new input dataset (also called the out of bag dataset) is added to all the bootstrap datasets, the decision from each bootstrap is recorded. The aggregated decision is the decision for the new input. This type of decision-making using a bootstrap dataset is called bagging [28,29].

The algorithm is used for binary classification and regression problems. RF is considered an accurate and robust method because of the number of decision trees constructed to provide a prediction for the new dataset. RF does not suffer from the overfitting problem. The random forest is a time-consuming algorithm because decisions are made from many trees [30,31,32].

### 3.4. K-Nearest Neighbours

The K-nearest neighbours (KNN) is a simple supervised, non-parametric algorithm used for classification and regression problems. For a data point x to be classified, its K-nearest neighbours are retrieved, as a neighbourhood of x. Voting among the neighbourhood data is usually used to decide the classification for the point x. The critical point is to select the value for the *k*. If the value of *k* is not appropriate, there is a low chance of obtaining promising results for any dataset. The algorithm becomes slow when we increase the number of k values [30,31].

### 3.5. Convolutional Neural Network (CNN)

CNN is a deep learning algorithm that is very helpful for image classification. The CNN model on image datasets generally takes an image as the input, and the output is the likelihood of the class to which it belongs. The critical layers in the CNN are the convolution layer and the max pooling layers. The input image is given to the convolution layer, and it applies different kernels/filters that extract the low-level features. The following are the fundamental building blocks on CNN [33].

#### 3.5.1. Stride

**Figure 3:**
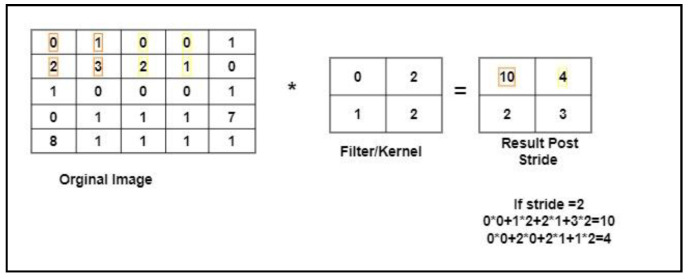
A Stride Example.

It is the number of picture elements the kernel/filter shifts over the input at a time. Figure 3 shows an example of how the stride process works. In the example, the filter convolved around the input by shifting two units at a time. To calculate the first block of the resulting image after a stride operation with stride = 2 was performed. We applied a cross product between the image pixel and the filter pixel as (0 × 0) + (1 × 2) + (2 × 1) + (3 × 2) = 10; such that the first number (in orange color) was the image pixel and second number (in blue color) was the filter pixel.

The image was of size 5 × 5 with a filter of size 2 × 2, and a stride value equal to 2. The size of the reduced image was calculated using Equation (6): where *n_in_* (in our case, 5) is the number of input features, *n_out_* is the number of output features, *k* is kernel size (in our case, 2), *p* is the convolution padding size (in our case, 0), and *s* is convolution stride size (in our case, 2). The resulting image was of size 2 × 2.

(6) $$n_{out}=\left|\frac{n_{in}+2p-k}{s}\right|+1$$

#### 3.5.2. Padding

**Figure 4:**
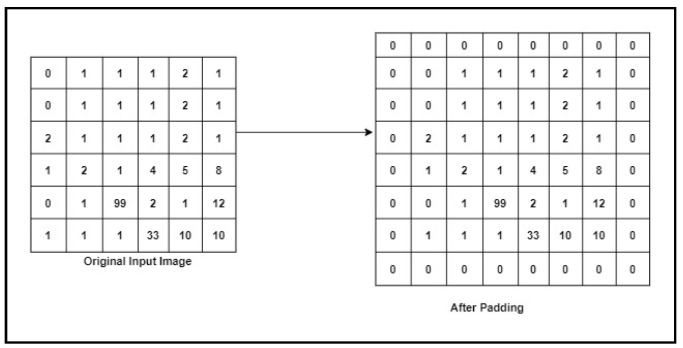
The Padding Example.

The convolution layer without padding does not preserve the spatial size of the input image. When the input is given to the convolution layer with padding, we add zeros to the border of input, which helps in extracting the features from the corner of the image. The input image dimensions in Figure 4 are 6 × 6 × 1, where 6 × 6 is the image size, and the number of channels is 1, which means the image has only one channel. If we set the padding size = 2, we get the image size of image 8 × 8 × 1. Figure 4 shows the original image and the matrix after applying the padding process.

#### 3.5.3. Max Pooling

**Figure 5:**
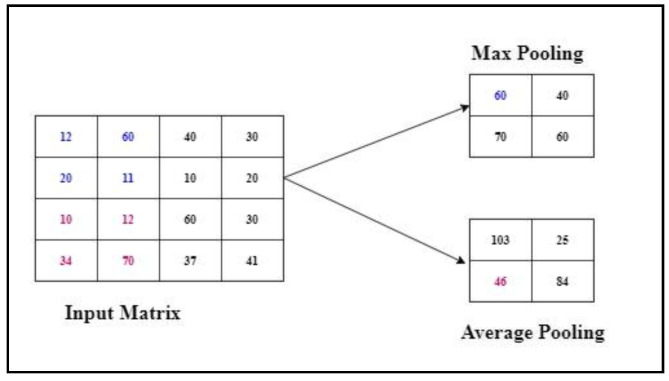
Max pooling.

The max pooling reduces the size of the feature maps. The advantage of using this layer is to reduce the computational power. Although the size of the image is reduced, the vital information is still maintained. There are different types of pooling, which are as follows max pooling and average pooling. Max pooling chooses the highest/topmost value from the image covered by the kernel. In contrast, average pooling calculates the midpoint/mean value from the portion of an image covered by the filter. An example is shown in Figure 5, where the input image is 4 × 4; if we apply stride = 2 and perform max pooling, the results are obtained in each block as the max value out of the selected block.

#### 3.5.4. Activation Function

The activation function is also called a transfer function, computes a weighted sum of the input and biases. The activation function decides if the weights and bias’ values will activate/fire the neuron. The activation function aims to convert a linear input signal of the model into non-linear output signals. The simple CNN model consists of an input layer hidden layer (having a convolution layer, activation, and max pooling layer) and the output layer. The activation function choice will help us perform classification or regression tasks when throwing the model’s output. After the hidden layer, the activation function is invoked to learn a non-linear form of linear mapping before making any predictions. Some activation functions are Rectilinear, sigmoid, SoftMax, and tanh [28].

- Sigmoid function: The sigmoid function exists between 0 and 1, and its shape looks like an S shape. Sigmoid is the correct choice when we have to predict the likelihood of a model. Equation (7) illustrates the sigmoid function. Since the sigmoid function is differentiable, the sigmoid function’s derivative is shown in Equation (8) to calculate the slope of the sigmoid curve.
  (7) $$\sigma \left(x\right)=\frac{1}{1+e^{-z}}$$
  (8) $$\sigma^{\prime }=\sigma \left(x\right)\left(1-\sigma \left(x\right)\right)$$
- Rectilinear function: The Rectilinear function, also called ReLU. It has values between 0 and infinity, and it provides better performance than the sigmoid function. Equation (9) shows the derivative of the ReLU function.
  (9) $$R\left(z\right)=\left\{\begin{array}{c} 1\;\;\;\;\;\;\;\;if\;z>0\\ 0\;\;\;\;\;\;\;\;if\;z<0 \end{array}\right\}$$

In one of the proposed models, we used the sigmoid activation function since our problem evolved to predict whether the candidate has autism or not, making it a binary classification.

## 4. The Proposed Hybrid Autoencoder-Based Classifier

**Figure 6:**
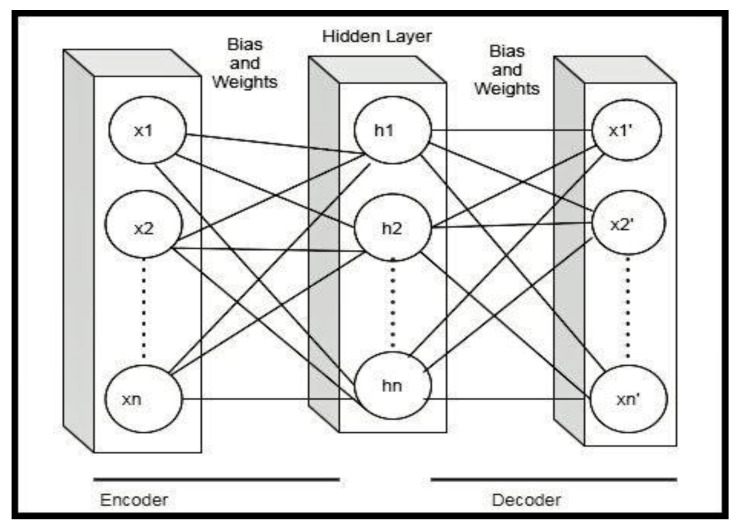
The autoencoder architecture.

Autoencoders play a vital role in extracting low dimensional features, and these features can be given to machine learning models or deep learning models to perform classification tasks. The architecture of autoencoders reduces dimensionality using non-linear optimization. Our proposed method focused on using the undercomplete autoencoder to extract useful information from the input layer by having fewer neurons in the hidden layer than the input. The architecture of an undercomplete autoencoder is shown in Figure 6. It is the simplest form of constructing an autoencoder by limiting the amount of information that can flow through the network. This can be achieved by reducing the number of neurons in the hidden layer. This helps to obtain essential features from the data. By penalizing the structure according to the reconstruction error, our architecture learns the most important input data attributes and show how to reconstruct the original input from an “encoded” state. 

**Figure 7:**
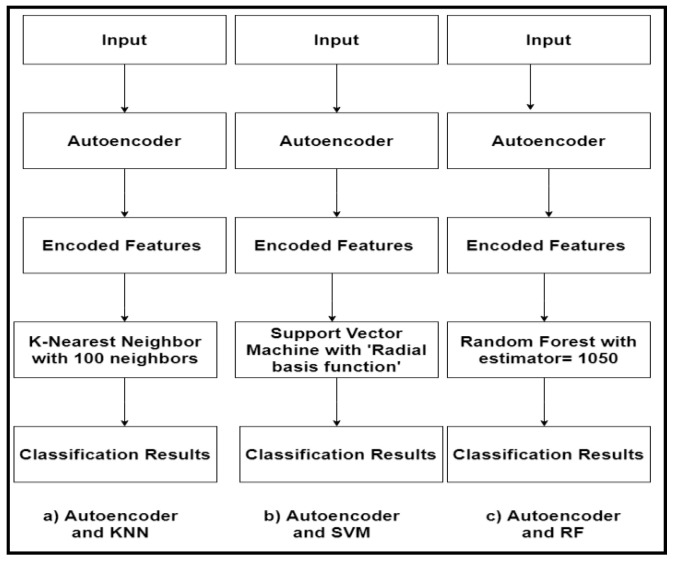
The hybrid autoencoder-based machine learning classifier. KNN: K-nearest neighbour; RF: random forest.

The input fMRI was fed to the Pearson’s correlation function to compute the pairwise correlation. This data was provided as input to an autoencoder, which will help extract lower-dimensional features (in our case, 4975 features from 9950) to send it to the classification models. In the paper, we focused on using four main classifiers, including KNN, SVM, RF, and CNN. Figure 7 shows the flowchart of combing the autoencoder and the adopted machine learning classifiers. In the autoencoder–KNN model, for calculating the distance between *k* points, the value of *p* was set to 2, which was for the Euclidean distance and 100 neighbours. For the autoencoder–SVM model, in the parameter list, we chose C = 2.0, which is a regularization parameter. For the autoencoder–RF model, we assigned the estimators value to 1050 and the depth value to 90.

**Figure 8:**
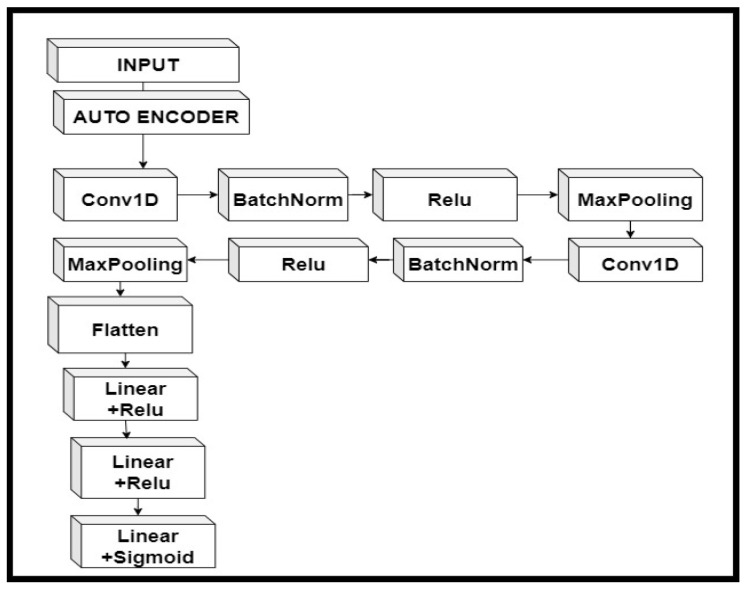
The hybrid model of an autoencoder and convolutional neural network (CNN).

In the proposed autoencoder–CNN, the CNN consisted of two 1D convolution layer followed by batch normalization, an activation function, and a max pooling layer. After flattening the CNN layer, it worked as a fully connected neural network, and we had two linear layers with an activation function. Since the output predicts whether an individual is diagnosed with autism or not, we applied the sigmoid activation function. Figure 8 shows the workflow of combining an autoencoder with CNN.

## 5. Experiment Results

**Figure 9:**
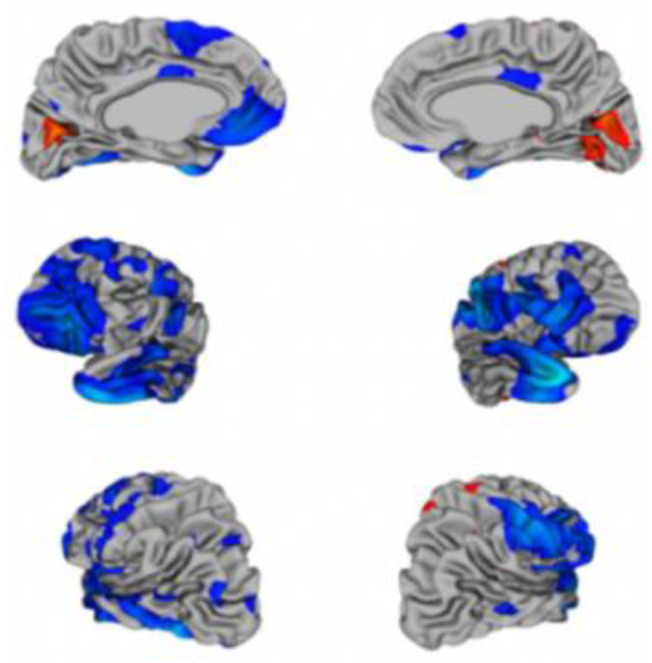
The brain structure variations in an autistic child.

The Google Colaboratory (Colab) was used to perform experiments, a free online cloud-based Jupyter notebook that allowed us to train our machine learning and deep learning models on CPUs, GPUs, and TPUs. The ABIDE-I dataset has rs-fMRI data for 1112 candidates, along with phenotypic information. This data is slice time corrected, motion-corrected, and normalized. In our study, all rs-fMRI data were from the CPAC preprocessing pipeline and band-pass filtered (0.01–0.1 Hz). From these 1112 subjects, 1035 subjects were considered for our study since only these subjects had completed phenotypic information. To limit the variance between outputs to just preprocessing, statistical derivatives for each pipeline and strategy were calculated using the CPAC software [8]. The rs-fMRI (shown in Figure 9) stands for resting-state fMRI, a type of functional magnetic resonance imaging (fMRI) used in brain mapping to evaluate regional interactions in a resting or task-negative state when an explicit task is not performed. The resting-state process helps explore the brain’s functional organization and examine if altered in neurological or mental disorders. Figure 9 shows the brain structure of a child having ASD [34].

### Experimental Analysis

To evaluate the performance, we used k-fold cross-validation. We used three machine learning algorithms and two forms of neural networks to detect ASD. Table 1 compares the accuracy, sensitivity, and specificity of the four proposed models with individual-based classifiers. The accuracy refers to the number of correct predictions made by the predictive model over the rest of the predictions. Sensitivity refers to how sensitive the classifier is in detecting positive instances, and specificity is the proportion of the true negatives correctly identified by a diagnostic test. The results show that an autoencoder, combined with CNN, had achieved 84.05% accuracy, which outperformed other methods. The percentage of improvement in each measure is illustrated in Table 2.

**Figure 10:**
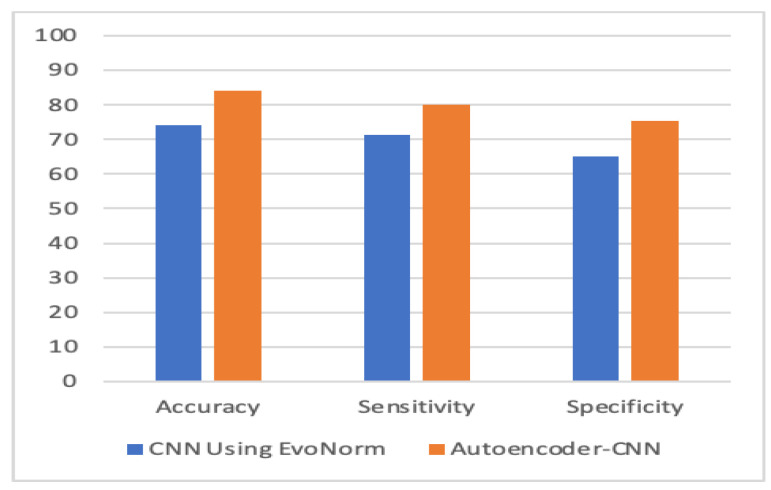
Autoencoder-CNN vs. CNN. CNN: convolution neural network.

**Figure 11:**
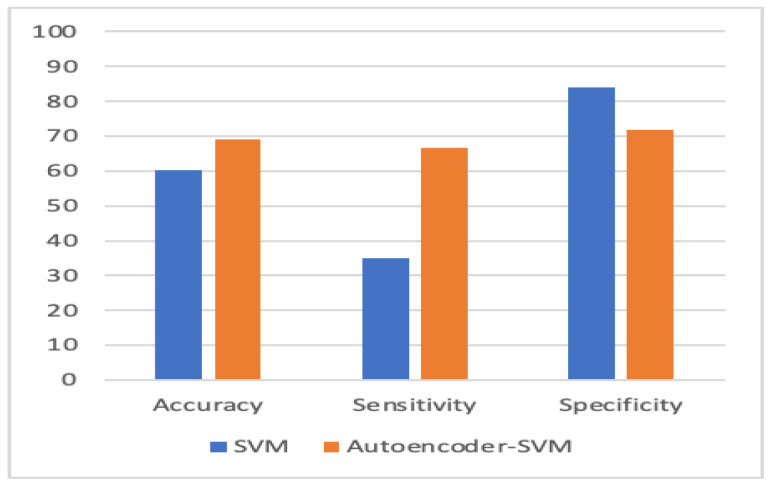
Autoencoder-SVM vs. SVM.

**Figure 12:**
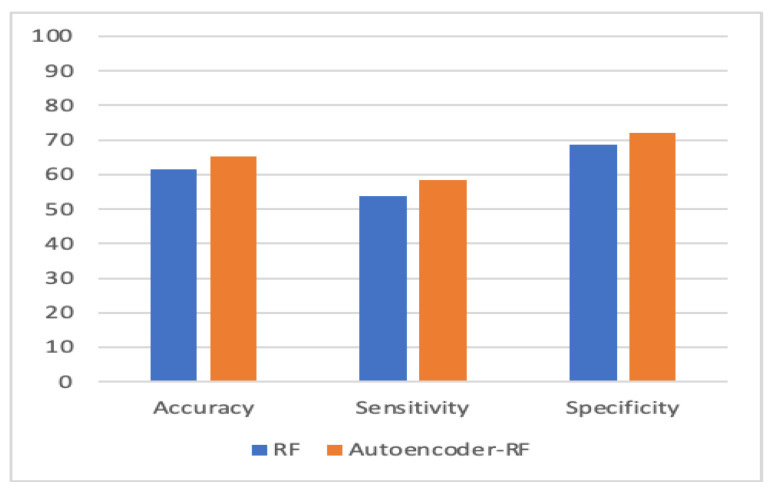
Autoencoder-RF vs. RF.

**Figure 13:**
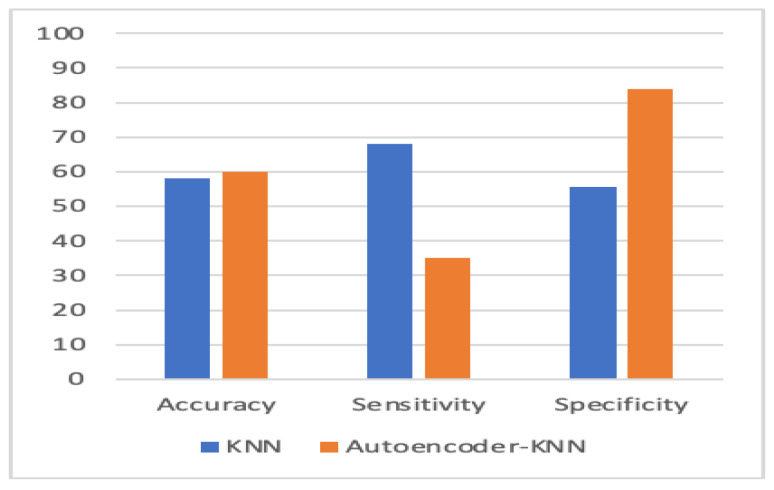
Autoencoder-KNN vs. KNN.

**Figure 14:**
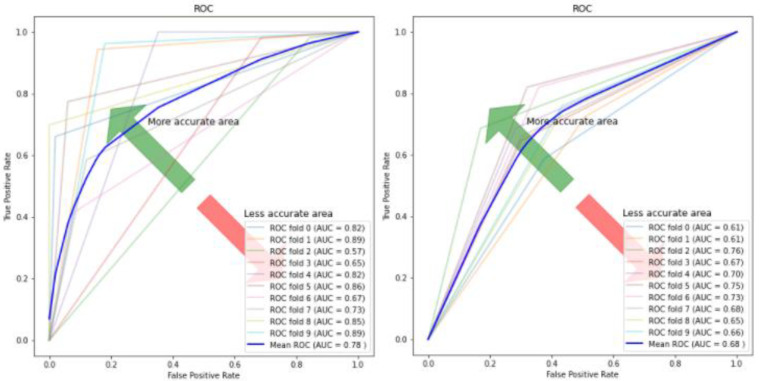
ROC curve and AUC values for autoencoder–CNN (**Left**) and CNN (**Right**). ROC: Receiver Operating Characteristics; AUC: Area under the Curve.

**Figure 15:**
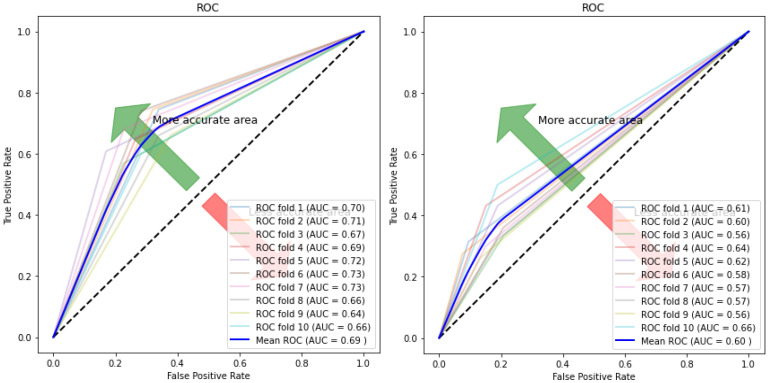
ROC curve and AUC values for autoencoder–SVM (**Left**) and SVM (**Right**).

**Figure 16:**
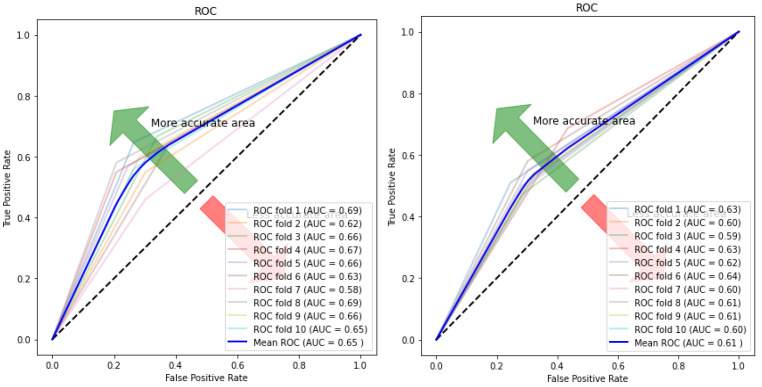
ROC curve and AUC values for autoencoder–RF (**Left**) and RF (**Right**).

**Figure 17:**
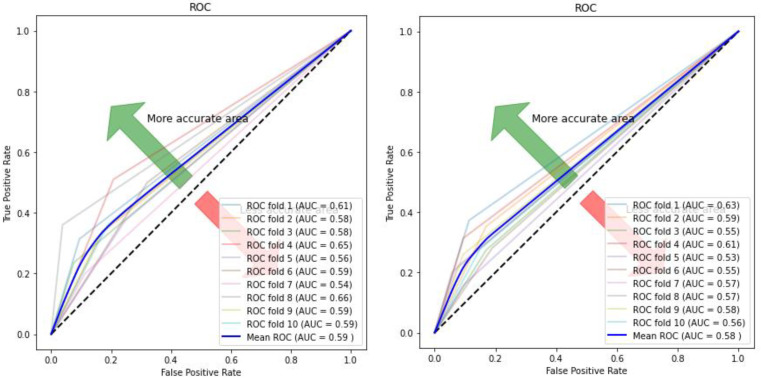
ROC curve and AUC values for autoencoder–KNN (**Left**) and KNN (**Right**).

Figure 10, Figure 11, Figure 12 and Figure 13 compare the performance of the proposed hybrid models against individual classifiers. We could observe that the hybrid autoencoder-based models outperformed the individual methods measured by the increased value of the accuracy, sensitivity, and the low values of the specificity measures. The Receiver Operating Characteristic (ROC) curve for each hybrid model, compared to the individual classifier, is shown in Figure 14, Figure 15, Figure 16 and Figure 17. We could observe that the ROC curve for autoencoder–CNN was better than any other model. ROC is a probability curve, and AUC represents the degree or measure of separability. The higher the AUC, the better the model was at the prediction.

Table 3 shows the values of accuracy, sensitivity, specificity, and AUC [35]. The first value indicates the average of all the 10 k-folds, and the second value is the standard deviation. The standard deviation helps to calculate the amount of variation in a set of values [36]. A low standard deviation indicates that the values tend to be close to the mean, while a high standard deviation indicates that the values are dispersed over a broader range [37,38].

**Figure 18:**
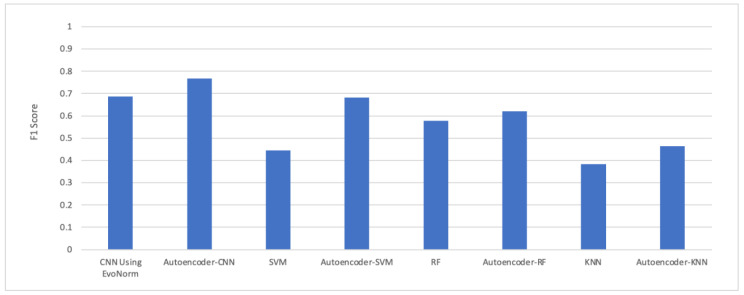
The F1 score.

The F1 score was also calculated for all the proposed models, as shown in Figure 18. CNN achieved better results compared to other models. The F1 score can be interpreted as a weighted average of precision and recall. The relative contribution of precision and recall to the F1 score are equal. The F1 score [39,40] is calculated as:

(10) F1=2×(precision×recall)/(precision+recall)

**Figure 19:**
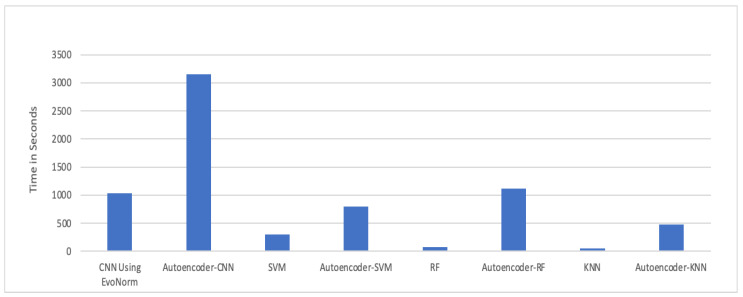
Computational time (s).

The experimental results in Figure 18 show that the autoencoder with CNN was the best performing hybrid model for our problem. The computational time taken by each model is illustrated in Figure 19. The autoencoder combined with CNN has the highest computational time for training, while the autoencoder–KNN had the lowest computational time.

## 6. Conclusions and Future Direction

The purpose of this paper was to provide an automatic diagnosis algorithm that classifies individuals with ASD and non-ASD. Experimental results show that the hybrid autoencoder with convolution networks gave a better performance by achieving 84.05% accuracy and an AUC value of 0.78. An autoencoder with CNN consumed approximately 55 min to train the model. The autoencoder performed feature selection, and those features can be given as input to any classification model. The AUC score for an autoencoder combined with SVM, random forest, and KNN was 0.69, 0.65, and 0.59, respectively. The main drawback of our experiment was the size of the dataset. We had a small dataset with 1112 rs-fMRI images. In the future, personal characteristics data like birth weight, mother’s age, and family history could give us more promising results. Survey data related to the child’s daily behaviour or a short clip of the participant could help detect autism, which can add more weightage to the number of features. Complex models like ResNet-50 can be applied to the data of fMRI through the approach of transfer learning. Future directions also include a combative analysis to compare the performance of other dimensionality reduction methods such as the principal component analysis, linear discriminant analysis (linear models), locally linear embedding, and Isomap (non-linear models) when combined with machine learning or deep learning classifiers. The results of KNN can be improved by using the Grid Search CV method, and it helps to loop through predefined hyperparameters and fit your estimator (model) on your training set. So, in the end, we could select the best parameters from the listed hyperparameters. In summary, we developed and compared an autoencoder’s performance combined with other models to the performance of the individual models to understand how much the emphasis of encoded features helps classify ASD candidates from fMRI images.

## Figures and Tables

**Table 1:** Sensitivity and specificity.

Model	Accuracy %	Sensitivity %	Specificity %
Autoencoder–CNN	84.05	80	75.3
Evo Norm CNN	74	71.33	65.2
SVM	60.2	35.1	84.1
Autoencoder–SVM	69.1	66.5	71.69
Random Forest (RF)	61.5	53.8	68.8
Autoencoder–RF	65.3	58.3	72.1
KNN	58.1	68.2	55.5
Autoencoder–KNN	60.1	35	84

CNN: convolution neural network; SVM: support vector machine; KNN: K-nearest neighbour.

**Table 2:** Percentage of improvement in accuracy, sensitivity, and specificity.

Model	Accuracy %	Sensitivity %	Specificity %
Autoencoder–CNN	10.05%	8.67%	10.1%
Autoencoder–SVM	8.9%	31.4%	−12.41%
Autoencoder–RF	3.8%	−0.5%	3.3%
Autoencoder–KNN	2%	−33.2%	28.5%

**Table 3:** Average and standard deviation in accuracy, sensitivity, specificity, and AUC.

Model	Accuracy %	Sensitivity %	Specificity %	AUC
KNN	0.582 (+/−) 0.02	0.682 (+/−) 0.07	0.555 (+/−) 0.02	0.58 (+/−) 0.02
Autoencoder–KNN	0.601 (+/−) 0.035	0.35 (+/−) 0.09	0.84 (+/−) 0.08	0.595 (+/−) 0.03
Evo Norm CNN	0.743 (+/−) 0.05	0.713 (+/−) 0.07	0.652 (+/−) 0.09	0.68 (+/−) 0.05
Autoencoder–CNN	0.84 (+/−) 0.07	0.8 (+/−) 0.19	0.753 (+/−) 0.22	0.78 (+/−) 0.11
RF	0.615 (+/−) 0.01	0.583 (+/−) 0.06	0.688 (+/−) 0.04	0.612 (+/−) 0.01
Autoencoder–RF	0.653 (+/−) 0.02	0.583 (+/−) 0.06	0.721 (+/−) 0.05	0.651 (+/−) 0.03
SVM	0.603 (+/−) 0.03	0.351 (+/−) 0.07	0.841 (+/−) 0.04	0.6 (+/−) 0.03
Autoencoder–SVM	0.691 (+/−) 0.03	0.665 (+/−) 0.06	0.716 (+/−) 0.06	0.69 (+/−) 0.03

AUC: Area under the Curve.
